# A New Regulatory Network Controls Chilling Injury in Peach Fruit by γ-Aminobutyric Acid

**DOI:** 10.3390/foods12040696

**Published:** 2023-02-06

**Authors:** Chunbo Song, Cheng Zhou, Yongjian Pan, Zhenfeng Yang

**Affiliations:** College of Biological and Environmental Sciences, Zhejiang Wanli University, Ningbo 315100, China

**Keywords:** γ-aminobutyric acid, peach fruit, cold tolerance, polyamines, proline

## Abstract

The control of chilling injury in peach fruit by a new regulator network, that exogenous γ-aminobutyric acid (GABA) regulates the metabolisms of polyamines (PAs), the GABA shunt, and proline, is still unclear. This study found that GABA induced an increase in the expression of *PpADC* and *PpODC* and a decrease in the expression of *PpPAO* expression, resulting in the accumulation of PAs. There was also an increase in the expression of *PpGAD*, which improved GABA content, and an increase in the expression of *PpP5CS* and *PpOAT*, which improved proline content. The correlation analysis showed that an increase in *PpADC*/*PpP5CS* expression was closely associated with the accumulation of putrescine and that the synergistic increase in the expression of *PpODC* and *PpGAD*/*PpP5CS*/*PpOAT* was closely related to the accumulation of spermine, proline, and GABA induced by GABA. Importantly, arginine and *PpADC* played a key role in putrescine accumulation, whereas ornithine and *PpODC*/*PpOAT* played a crucial role in the synergistic accumulation of spermine, proline, and GABA induced by GABA. This study provides new information on GABA-induced cold tolerance in peach fruit.

## 1. Introduction

Low temperature (LT) storage is a common and efficient way to keep fruits and vegetables fresh. Appropriate LT can significantly delay fruit tissue senescence, inhibit the growth of pathogenic microorganisms, and extend shelf life [1,2]. Currently, there has been extensive research into the mechanisms of and controls for chilling injury (CI) in peach fruit under LT storage, such as γ-aminobutyric acid (GABA) [3], oxalic acid [4], melatonin [5], 2,4-epibrassinolide [6], methyl jasmonate [7], ethylene [8], nitric oxide (NO) [9], and glycine betaine [10], which can effectively improve cold tolerance in peach fruit.

GABA is a plant signaling molecule that regulates the pH in and among cells, balances the nutrition of carbon and nitrogen, and contributes to plants’ resistance to adverse environments [11]. Studies have shown that GABA can actually postpone the appearance of CI in banana fruits [12], citrus fruits [13], blueberry fruits [14], Nanguo pears [15], Chinese olive fruits [16], ‘Sahebi’ grapes [17], and aonla fruits [18] and maintain their good quality and shelf life. When plants react to LT, dark surroundings, or mechanical damage, the content of endogenous GABA increases rapidly [19]. GABA is generated directly from glutamate acid decarboxylase (GAD) in the GABA shunt pathway [20].

Polyamines (PAs) metabolism and proline metabolism are closely related to the GABA shunt. Some amino acids act as common precursors, establishing a close link with a variety of metabolic pathways. PAs are involved in controlling the tolerance of abiotic stress in plants [21], including putrescine (Put), spermidine (Spd), and spermine (Spm). Ornithine and arginine are indirectly decarboxylated under the catalysis of ornithine decarboxylase (ODC) and arginine decarboxylase (ADC), respectively, to produce Put [22], and Put is added to aminopropyl to synthesize Spd and Spm [23]. PAs degradation is catalyzed by diamine oxidase (DAO) and polyamine oxidase (PAO) [21,24]. Put can be converted into GABA under the catalysis of DAO [21,25], and PAO is the key enzyme for the degradation of Spd and Spm.

Proline, as a soluble osmotic substance, can maintain cellular osmotic equilibrium and protect the subcellular structure, the content of which is closely linked to the cold sensitivity of post-harvest fruits and vegetables [26]. Glutamic acid is a common precursor for the synthesis of proline and GABA. It is indirectly reduced to proline under the catalyst Δ1-pyrroline 5-carboxylate synthetase (P5CS). Ornithine is a common precursor substance for the synthesis of proline and PAs, and is indirectly reduced to proline under the catalysis of ornithine aminotransferase (OAT) [27]. Additionally, glutamic acid is indirectly produced from proline through the catalysis of proline dehydrogenase (PDH) [28].

Therefore, PAs metabolism, the GABA shunt, and proline metabolism play a crucial role in plants’ response to stress. At present, the correlative mechanisms of PAs metabolism, the GABA shunt, and proline metabolism have been reported in GABA-induced disease resistance in apples [29], NO-induced cold tolerance in banana fruit [30], PSKα-induced cold tolerance in banana fruit [31], and melatonin-induced cold tolerance in cucumber fruit [32]. It has been confirmed that PAs metabolism, the GABA shunt, and proline metabolism are involved in regulating melatonin-induced cold tolerance in peach fruit [5]. It is well known that the application of exogenous GABA induces the cold tolerance of fruits by regulating the levels of PAs and proline and maintains the good quality of post-harvest horticultural products [29,33]. However, relevant studies have not been reported in peach fruit. To this end, it is very important to explore the mechanism of the GABA shunt, PA metabolism, and proline metabolism in cold tolerance induced by GABA in peaches. The purpose of this study was to investigate the effects of GABA treatment on the GABA shunt, PAs metabolism, and proline metabolism in post-harvest peaches.

## 2. Materials and Methods

### 2.1. Materials and Treatments

Commercially mature (around 120 days after flowering) “Hujing” peaches (*Prunus persica* L. Batsch) were harvested from Fenghua District, Ningbo City, Zhejiang Province and transported to the laboratory within 1 h. Evenly sized and mature peaches were randomly divided into two groups, each with 100 fruits. The peaches were dipped into either a GABA solution (5 mmol·L^−1^) or a water solution for 20 min, creating the GABA treatment group (GABA) and the control group (CK), respectively. The peaches were naturally dried by air and stored in an incubator (4 ± 1 °C), which was sampled every 7 days in order to determine the physiological indicators and key gene expression.

### 2.2. Determination of Fruit Firmness

Six peaches from each treatment group were randomized, and a TMS-Touch Full Touch Properties analyzer was used to test the TPA of equatorial symmetrical parts peeled from the peaches in different treatment groups. The test program parameters included the probe diameter 7.5 mm, test speed 30 mm/min, deformation 15%, tripping force 0.1 N (sensor range 25 N), or 1 N (sensor range 1000 N). The output of firmness is represented by a double-crested curve, where the maximum F value for the first peak is taken as the firmness data and reported in units of N.

### 2.3. Determination of Fruit Color

Six peaches from each treatment group were randomized. A CR410 color difference meter was used to take three points around the equatorial area of the peeled fruits in order to measure the value of flesh a and b. The colorimeter must be calibrated with a standard color plate prior to use. The Hue angle (h°) was calculated according to the formula Hue angle = arctan (b/a). This value represents the comprehensive chromaticity index, with a value range of 0–180. As the value increases, the color changes from amaranth, red, orange, yellow, yellow-green, green, and blue-green. When the value is 90, the color is yellow.

### 2.4. Determination of Total Soluble Solids (TSS)

Six peaches were randomly chosen from each treatment group. A small amount of flesh was taken from the symmetric part close to the equator of the fruit. After the juice was compressed, it was completely mixed. The content of the TSS was determined immediately using a handheld digital saccharimeter.

### 2.5. Determination of Percentage of Extractable Juice

Six peaches were randomly chosen from each treatment group. A small amount of fruit flesh was taken from the symmetrical part near the equator of the fruit, sliced into small pieces weighing 10 g (6 mm in diameter and 6 mm thick) with a blade, and placed in a centrifuge tube that was previously weighed with absorbent cotton. Then, the small pieces were weighed and centrifuged at 8000 rpm for 10 min. The fragments of peach tissue were removed and weighed again. The percentage of extractable juice is calculated as the rate of weight loss of the fruit after centrifugation.

### 2.6. Determination of Polyamine Content

The samples of ground peaches with liquid nitrogen were weighed and mixed with pre-cooled perchloric acid in an ice bath at 37 °C for 1 h. After centrifugation, the supernatant was mixed with NaOH and benzoyl chloride, which then reacted in a water bath at 37 °C for 30 min. The reaction was immediately stopped by adding a saturated NaCl solution. The pre-collated ether was added for extraction. After centrifugation, the ether stage was collected, dried with nitrogen, and dissolved by methanol chromatography. The above samples after filtration were used to determine the PAs content.

High Performance Liquid Chromatography (HPLC, Waters 2695, Waters Corporation, Milford, MA, USA) with a model 2998 photodiode array detector (DAD, Waters) and a 4.6 × 150 mm C18 column was used to detect and analyze the PAs at 230 nm. The mobile phase consisted of 64% (*v/v*) chromatographic methanol with a flow rate of 0.8 mL/min. The column temperature was 30 °C and a sample injection volume of 20 µL was used.

### 2.7. Determination of GABA Content

The peach sample ground with liquid nitrogen was weighed, mixed with lanthanum chloride, and vortexed at room temperature for 15 min. After centrifugation, the supernatant was mixed with KOH. After centrifugation, the supernatant was mixed with KOH, phosphatic buffer, phenol, and sodium hypochlorite. Then, the mixture was reacted in a boiling water bath for 10 min and immediately placed in ice to cool for 5 min. The above blended solution was blended with 60% ethanol and measured at 645 nm with the Shimadzu UV-1750 ultraviolet spectrophotometer in order to calculate the GABA content.

### 2.8. Determination of Proline Content

The peach sample ground with liquid nitrogen was weighed and reacted with sulfosalicylic acid in a boiling water bath for 10 min. The supernatant was reacted with glacial acetic acid and ninhydrin color developing solution in a boiling water bath for 40 min. After cooling to room temperature, toluene was added for extraction and the absorbance was measured at 520 nm in order to calculate the proline content.

### 2.9. Total RNA Preparation and cDNA Synthesis

A plant RNA kit (Omega Bio-Tek, Inc., Norcross, GA, USA) was used to prepare the total RNA. A NanoDrop 2000 spectrophotometer was used for RNA quantification. A SuperRT First Strand cDNA Synthesis Kit (CWBIO, Beijing, China) was used for cDNAs synthesis. A SYBR Green PCR master mix (Thermo Fisher Scientific, Inc., Pittsburgh, PA, USA) was used for quantitative reverse transcription polymerase chain reaction (qRT-PCR) analyses.

The transcription levels of the key enzyme genes, including *PpOAT*, *PpP5CS*, *PpPDH*, *PpADC, PpOD*C, *PpPAO*, and *PpGAD*, of proline, PAs, and GABA shunt metabolism were determined, and information regarding the primes was determined as previously reported [5].

### 2.10. Statistical Analysis

The study was entirely randomized and replicated at least three times. Data are expressed as means ± standard errors (SE). SPSS version 16.0 (SPSS, Inc., Chicago, IL, USA) was used for the statistical analysis. Asterisks (*) indicate significant differences between the GABA and CK groups. Student’s unpaired *t*-test; (*) *p* < 0.05, (**) *p* < 0.01. An analysis of correlation was carried out with Omicroshare Tools.

## 3. Results

### 3.1. Effect of Exogenous GABA Treatment on Quality Parameters

**Figure 1:**
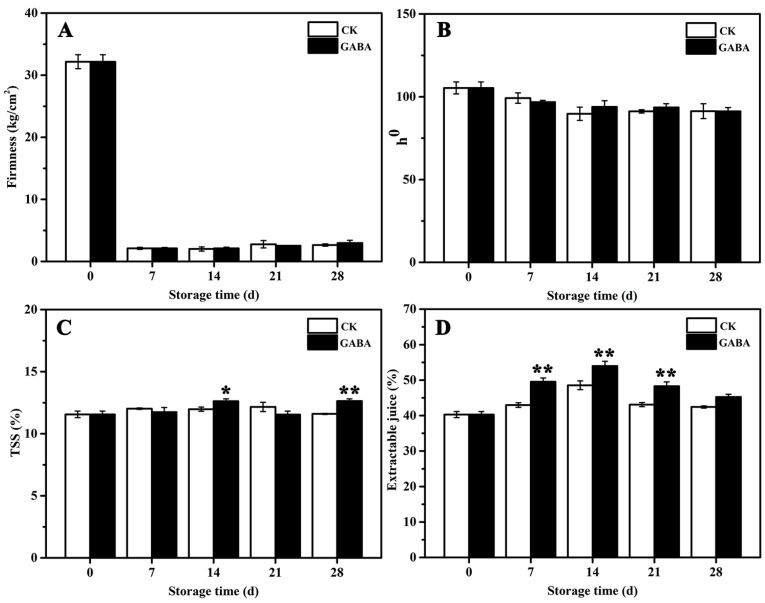
Effect of exogenous GABA on peach fruit quality. (**A**) Firmness, (**B**) L*, (**C**) TSS, and (**D**) extractable juice. All data are expressed as means ± standard errors (SE). Asterisks (*) indicate significant differences between the GABA and CK groups. Unpaired Student’s *t*-test; (*) *p* < 0.05, (**) *p* < 0.01.

Changes in the quality parameters for the peach fruits in the CK and GABA groups are shown in Figure 1. The firmness of peaches stored for 7 days declined significantly, and no significant differences were found between the treatments during storage (Figure 1A). No significant differences in the hue angle (h°) of the peach color were observed between the treatments throughout the storage process (Figure 1B). Compared to the CK group, the GABA-treated peaches maintained significantly higher TSS levels on days 14 and 28 (Figure 1C), and retained significantly higher extractable juice before days 28 (Figure 1D).

### 3.2. Effect of Exogenous GABA Treatment on Polyamines Metabolism

**Figure 2:**
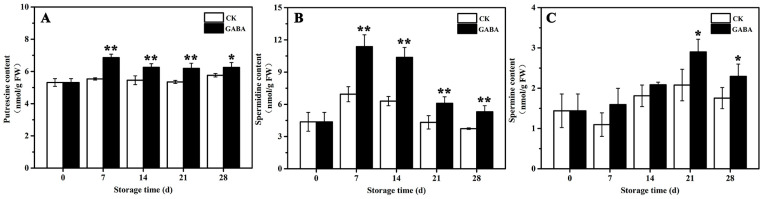
Change in (**A**) putrescine, (**B**) spermidine, and (**C**) spermine in peach fruit induced by GABA. All data are expressed as means ± standard errors (SE). Asterisks (*) indicate significant differences between the GABA and CK groups. Unpaired Student’s *t*-test; (*) *p* < 0.05, (**) *p* < 0.01.

The mechanism of PAs metabolism, which is involved in regulating GABA-induced cold tolerance in peach fruit, was investigated. As shown in Figure 2, in the CK group, Put content tended to be flat during the entire storage period (Figure 2A), Spd content increased first and then decreased (Figure 2B), and Spm content decreased first and then increased (Figure 2C). In the GABA group during the entire storage period, Put content increased first and then tended to be flat (Figure 2A), Spd content first increased and then decreased (Figure 2B), and Spm content increased first and then decreased (Figure 2C). Compared to the CK group, GABA-treated peaches maintained significantly higher Put and Spd levels throughout storage, as well as a significantly higher Spm content on days 21 and 28.

**Figure 3:**
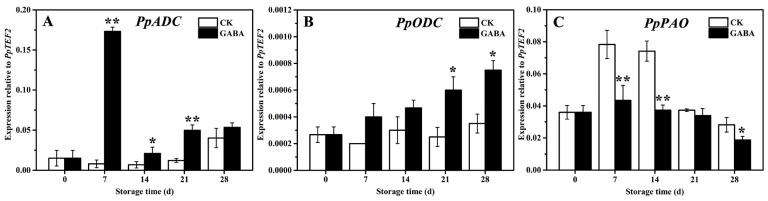
Change in the expression of (**A**) *PpADC*, (**B**) *PpODC*, and (**C**) *PpPAO* in peach fruit induced by GABA. All data are expressed as means ± standard errors (SE). Asterisks (*) indicate significant differences between the GABA and CK groups. Unpaired Student’s *t*-test; (*) *p* < 0.05, (**) *p* < 0.01.

*PpADC*, *PpODC*, and *PpPAO* are key enzyme genes in PAs metabolism in peach fruit. As shown in Figure 3, within the CK group throughout the storage period, the expression of *PpADC* tended to be flat and then increased on days 28 (Figure 3A), the expression of *PpODC* tended to be flat (Figure 3B), and the expression of *PpPAO* initially increased first and then decreased (Figure 3C). In the GABA group during the entire storage period, the expression of *PpADC* expression increased first, then decreased, and then increased (Figure 3A), the expression of *PpODC* increased progressively (Figure 3B), and the expression of *PpPAO* initially increased slowly and then decreased (Figure 3D). Compared with the CK group, GABA significantly increased the expression of *PpADC* on days 7, 14, and 21, as well as the expression of *PpODC* on days 21 and 28, and significantly downregulated the expression of *PpPAO* on days 7, 14, and 28.

### 3.3. Effect of Exogenous GABA Treatment on GABA Shunt

**Figure 4:**
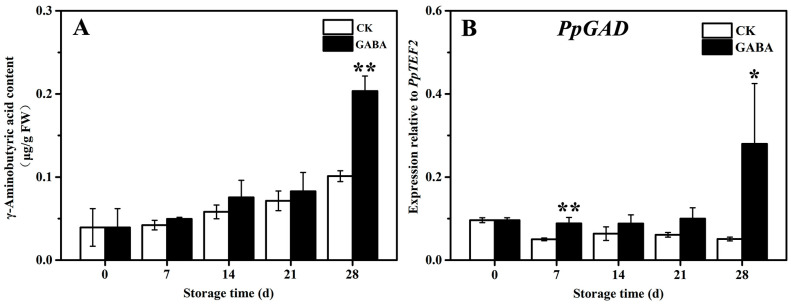
Change in (**A**) GABA content and (**B**) *PpGAD* expression in peach fruit induced by GABA. All data are expressed as means ± standard errors (SE). Asterisks (*) indicate significant differences between the GABA and CK groups. Unpaired Student’s *t*-test; (*) *p* < 0.05, (**) *p* < 0.01.

The mechanism of the GABA shunt involved in the regulation of GABA-induced cold tolerance in peach fruit was investigated. As shown in Figure 4, the content of GABA in the CK and GABA groups grew progressively, and the content of GABA in the GABA group was significantly higher than that of the CK group (Figure 4A). In the CK group throughout the storage period, the expression of *PpGAD* initially declined and then tended to be flat. In the GABA group during the entire storage period, the expression of *PpGAD* tended to be flat and then increased on days 28. Compared to the CK group, GABA considerably increased the expression of *PpGAD* on days 7 and 28 (Figure 4B).

### 3.4. Effect of Exogenous GABA Treatment on Proline Metabolism

**Figure 5:**
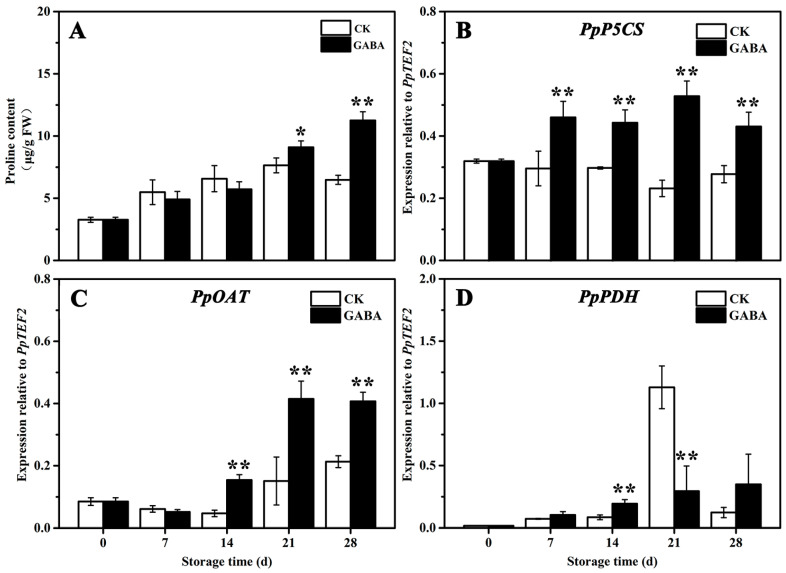
Change in (**A**) proline content and the expression of (**B**) *PpP5CS*, (**C**) *PpOAT*, and (**D**) *PpPDH* in peach fruit induced by GABA. All data are expressed as means ± standard errors (SE). Asterisks (*) indicate significant differences between the GABA and CK groups. Unpaired Student’s *t*-test; (*) *p* < 0.05, (**) *p* < 0.01.

The mechanism of proline metabolism involved in regulating GABA-induced cold tolerance in peaches was studied. As shown in Figure 5, the proline levels in the CK and GABA groups increased gradually, but the proline levels in the GABA group were significantly higher than that of the CK group on days 21 and 28 (Figure 5A). In the CK group throughout the storage period, the expression of *PpP5CS* tended to be flat (Figure 5B), the expression of *PpOAT* initially decreased slowly and then increased (Figure 5C), and the expression of *PpPDH* initially increased slowly, increased significantly on day 21, and then declined significantly on days 28 (Figure 5D). In the GABA group for the entire storage period, the expression of *PpP5CS* initially increased significantly and then tended to be flat (Figure 5B), and the expression of *PpOAT* and *PpPDH* gradually increased (Figure 5C,D). Compared with the CK group, GABA considerably improved the expression of *PpP5CS* throughout storage, as well as the expression of *PpOAT* on days 14, 21, and 28, and the expression of *PpPDH* on day 14, but significantly decreased the expression of *PpPDH* on day 21.

### 3.5. Correlation Analysis among Polyamines Metabolism, the GABA Shunt and Proline Metabolism

**Figure 6:**
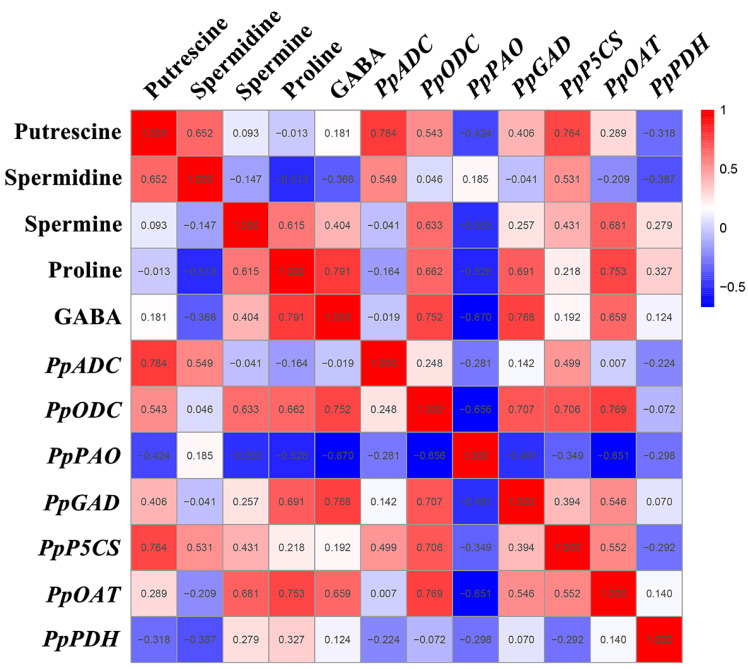
Thermal map analysis of the correlation of gene expression and PA, GABA, and proline content. The number represents the correlation coefficient, the red ground represents positive correlation, and the blue ground represents negative correlation.

A Pearson correlation analysis was performed in order to further investigate the mechanism of the GABA shunt, PAs metabolism, and proline metabolism in the regulation of GABA-induced cold tolerance in peach fruit. As shown in Figure 6, the correlation analysis between the metabolites and gene expression showed that Put content was significantly positively correlated with *PpADC*/*PpP5CS* expression (r = 0.7–0.8, *p* < 0.05) and was positively correlated with *PpODC* expression (r = 0.543, *p* < 0.05). The proline and GABA levels were significantly positively correlated with *PpODC*, *PpGAD*, and *PpOAT* expression (r = 0.6–0.8, *p* < 0.05). The content of GABA was significantly negatively correlated with *PpPAO* expression (r = −0.670, *p* < 0.05), and the level of proline was negatively correlated with *PpPAO* expression (r = −0.528, *p* < 0.05). The Spm content was significantly positively correlated with *PpODC*/*PpOAT* expression (r = 0.6–0.7, *p* < 0.05) and was negatively correlated with *PpPAO* expression (r = −0.503, *p* < 0.05). The Spd content was positively correlated with *PpADC* expression (r = 0.549, *p* < 0.05) and *PpP5CS* expression (r = 0.531, *p* < 0.05).

The metabolite correlation analysis found a significant positive correlation between Put content and Spd content (r = 0.652, *p* < 0.05), between proline content and Spm content (r = 0.615, *p* < 0.05), and between proline content and GABA content (r = 0.791, *p* < 0.05). The correlation analysis of gene expression showed that *PpODC* expression was significantly positively correlated with *PpGAD*/*PpP5CS/PpOAT* expression (r = 0.7–0.8, *p* < 0.05), and that *PpPAO* expression was significantly negatively correlated with *PpODC*/*PpOAT* expression (r = −0.7–−0.6, *p* < 0.05). *PpOAT* expression was positively correlated with *PpGAD* expression (r = 0.546, *p* < 0.05) and *PpP5CS* expression (r = 0.552, *p* < 0.05).

## 4. Discussion

GABA is a natural, safe, and bio-active compound. GABA accumulates rapidly when plants respond to external stress [11]. In addition, GABA has a variety of signal transduction functions, playing a pivotal role in plant growth and development, cell osmotic regulation, cell nitrogen supply, free radical sweeping, and biological and abiotic stress conditions [34]. GABA is useful for human health and is widely used in food [35]. In particular, GABA helps to regulate the post-harvest physiological process of fruits and vegetables and to maintain the post-harvest quality of fruits and vegetables [36]. For example, GABA treatment maintains high levels of chitinase, b-1,3-glucanase, phenylalanine ammonialyase, peroxidase, and polyphenol oxidase activities in pear fruits in order to withstand pathogen damage and to reduce the rate of decay [37]. 1-Methylcyclopropene (1-MCP) and GABA can effectively maintain post-harvest acidity and storage quality for apples and citrus fruits [38]. Exogenous GABA treatment can be directly involved in regulating malic acid metabolism, keeping organic acid levels in ‘Crisps Pink’ apples, and maintaining good flavor [38]. Exogenous GABA is involved in regulating ethylene anabolism, polyamine metabolism, and the GABA shunt in order to maintain fruit quality in “Golden Delicious” apples [33]. GABA + CaO composite treatment can improve the quality attributes and lifespan of fresh pistachios with shells in cold storage by decreasing the activity of polyphenol oxidase (PPO) in the shell of pistachios, whole fruit respiratory frequency, and microbial activity, and maintaining seed antioxidant activity, anthocyanin content in the seed shell, and phenol in the pistachio shell [39]. In this study, exogenous GABA treatment maintained higher levels of TSS on days 14 and 28 and higher extractable juice before days 28, leading to the preservation of peach quality (Figure 1C,D). Similar results were observed in melatonin-treated peach fruits [5].

The GABA shunt, PAs metabolism, and proline metabolism have been associated with regulating fruit disease resistance and cold tolerance. For example, exogenous GABA regulated the GABA shunt, active oxygen, and PAs metabolism in order to enhance apple disease resistance by enhancing the expression of *MdMT*, *MdMS*, *MdSAMS*, *MdSAMDC*, *MdODC*, *MdADC*, and *MdSPDS* and decreasing the expression of *MdPAO* and *MdDAO* [29]. NO improves the cold tolerance of banana fruit by enhancing ADC and ODC activities, which promotes PAs accumulation, increasing DAO, PAO, and GAD activities and reducing the activity of GABA transaminase, which promotes GABA accumulation, and enhancing OAT activity, which increases proline content [30]. PSKα maintains the quality of banana fruit and delayes the occurrence of banana CI by increasing ADC and ODC activities and decreasing DAO and PAO activities, which promotes the accumulation of Put, Spm, and Spd, as well as an increase in proline and GABA content [31]. Melatonin treatment improves the cold tolerance of cucumber fruit by increasing ADC and ODC activities and *CsADC* and *CsODC* expression, which promotes PA accumulation, enhancing P5CS and OAT activities, as well as *CsOAT* and *CsP5CS* expression, decreasing PDH activity in order to increase proline content, and enhancing GABA-T and GAD activities and *CsGAD* expression in order to improve GABA content [32]. Recent research has shown that exogenous melatonin treatment maintains the extractable juice and TSS of peach fruit in order to keep their quality and improves cold tolerance in peach fruit by increasing the expression of *PpADC*, *PpODC*, and *PpGAD* in order to increase GABA and PAs content, enhancing the expression of *PpP5CS* and *PpOAT*, and decreasing *PpPDH* expression in order to increase proline content [5]. In this study, GABA treatment improved cold tolerance in peach fruit by increasing the expression of *PpADC* and *PpODC* and decreasing the expression of *PpPAO* in order to enhance Put, Spd, and Spm content (Figure 2 and Figure 3), enhancing *PpGAD* expression in order to increase GABA content on days 28 (Figure 4), and decreasing *PpPDH* expression on days 21 and increasing *PpP5CS* and *PpOAT* expression in order to improve proline content (Figure 5).

GABA treatment maintains the quality of many horticulture crops and improves the cold tolerance of fruits by promoting the synergistic accumulation of metabolites or by promoting the synergistic expression of key genes in order to induce the synergistic accumulation of important metabolites. In the latest research report, it was found that GABA induced the synergistic accumulation of photosensitive choline (PC), diacylglycerol (DAG), and triacylglycerol (TAG) components in order to maintain the stability and fluidity of cell membranes and improves the cold tolerance of peach fruit. In addition, GABA induced the synergistic downward regulation of genes associated with phospholipid degradation and the biosynthesis of phospholipid acid in order to inhibit the accumulation of phospholipid acid and maintain a high level of phospholipids, thereby enhancing the cold tolerance of peaches [3]. In this study, the correlation mechanism of the GABA shunt, PAs metabolism, and proline metabolism in GABA-induced cold tolerance in peach fruit was analyzed. Significant positive correlation was found between the Put content and the Spd content and between the proline content and the Spm/GABA content (r = 0.6–0.8, *p* < 0.05) (Figure 6), showing that GABA induced the synergistic accumulation of PAs, proline, and GABA, further indicating that the GABA shunt, PAs metabolism, and proline metabolism were implicated in and closely related to the regulation of GABA-induced cold tolerance in peaches. Significant negative correlation was found between the GABA content and *PpPAO* expression (r = −0.670, *p* < 0.05) (Figure 6), which indicated that *PpPAO* was not the key gene for the degradation of polyamines in order to generate GABA. However, a significant positive correlation was found between the Put content and *PpADC*/*PpP5CS* expression, between the proline content and *PpODC*/*PpGAD*/*PpOAT* expression, between the GABA content and *PpODC*/*PpGAD*/*PpOAT* expression, between the Spm content and *PpODC*/*PpOA*T expression, and between *PpODC* expression and *PpGAD*/*PpP5C*S/*PpOA*T expression (r = 0.6–0.8, *p* < 0.05) (Figure 6), indicating that GABA induced an increase in the expression of *PpADC*/*PpP5CS* in order to contribute to Put accumulation, an increase in the expression of *PpODC*/*PpGAD*/*PpOAT* in order to contribute to the accumulation of proline and GABA content, an increase in the expression of *PpODC*/*PpOAT* in order to contribute to Spm accumulation, and a synergistic increase in the expression of *PpODC* and *PpGAD*/*PpP5CS*/*PpOAT* in order to contribute to an increase in Spm, proline, and GABA content. Importantly, arginine was a crucial substrate and *PpADC* was a crucial gene for GABA-induced Put accumulation, whereas ornithin was a key substrate and *PpODC*/*PpOAT* were key genes for the GABA-induced synergistic accumulation of proline, GABA, and Spm, especially *PpODC*.

## 5. Conclusions

This study revealed that GABA improved the cold tolerance of peaches by regulating the GABA shunt, PAs metabolism, and proline metabolism. GABA treatment increased the expression of *PpADC* and *PpODC* and decreased the expression of *PpPAO* in order to increase PAs content. Furthermore, GABA treatment increased *PpGAD* expression in order to improve the GABA content and enhanced *PpP5CS* and *PpOAT* expression in order to increase the proline content. In addition, arginine and *PpADC* played a key role in GABA-induced Put accumulation, whereas ornithine and *PpODC*/*PpOAT* played a crucial role in the GABA-induced synergistic accumulation of proline, GABA, and Spm. This research provided a theoretical scientific basis for amino acid metabolism and revealed the important key genes in the GABA shunt, PAs metabolism, and proline metabolism that are involved in regulating GABA-induced cold tolerance in peach fruit. Thus, this study lays the basis for further research into the functional roles of the key genes in the GABA shunt, PA metabolism, and proline metabolism.

## Data Availability

The data presented in this study are available on request from the corresponding author.
